# Germline *AIP* Testing in Pituitary Neuroendocrine Tumors: Diagnostic Yield, Variant Interpretation, and a Phenotype-Driven Clinical Approach—A Narrative Review

**DOI:** 10.3390/diagnostics16213471

**Published:** 2026-10-24

**Authors:** Cristiana Călin, Iulia Florentina Burcea, Ramona Dobre, Cătălina Poiană

**Affiliations:** 1 Department of Endocrinology, Carol Davila University of Medicine and Pharmacy, 020021 Bucharest, Romania; iulia.burcea@umfcd.ro (I.F.B.); ramona.dobre@umfcd.ro (R.D.); endoparhon@gmail.com (C.P.); 2 First Endocrinology Department, C.I. Parhon National Institute of Endocrinology, 011863 Bucharest, Romania

**Keywords:** AIP, pituitary neuroendocrine tumor, germline AIP testing, familial isolated pituitary adenoma, acromegaly

## Abstract

Germline pathogenic variants in the aryl hydrocarbon receptor-interacting protein (AIP) gene are the most common identifiable genetic cause of familial isolated pituitary adenomas (FIPA) and are particularly associated with young-onset growth hormone-secreting pituitary neuroendocrine tumors (PitNETs). This review critically evaluates current evidence on patient selection for germline AIP testing, variant interpretation, and emerging multigene approaches. A narrative PubMed/MEDLINE search identified English-language studies published between January 2006 and April 2025, including original clinical studies, international cohorts, systematic reviews, consensus statements, and relevant guidelines. Evidence was synthesized with emphasis on diagnostic yield, genotype–phenotype correlations, clinical indications for testing, and contemporary American College of Medical Genetics and Genomics/Association for Molecular Pathology (ACMG/AMP) variant classification. The highest diagnostic yield is consistently observed in FIPA, pituitary gigantism, childhood- or young-onset growth hormone (GH)-secreting macroadenomas, and selected patients with aggressive or otherwise high-risk phenotypes. Contemporary ACMG/AMP classification has lowered and refined estimates of clinically actionable AIP variants compared with historical studies. Targeted AIP testing remains appropriate for characteristic phenotypes, whereas multigene panels may be useful when syndromic features, atypical presentations, or persistent suspicion of hereditary disease remain after negative AIP testing. Current evidence supports phenotype-driven rather than universal germline AIP testing in PitNETs. Integrating age at onset, family history, tumor subtype, tumor characteristics, and standardized variant interpretation may maximize diagnostic yield while limiting unnecessary testing.

## 1. Introduction

Pituitary neuroendocrine tumors (PitNETs) are predominantly sporadic neoplasms; however, a small but clinically important proportion occurs in a hereditary setting, either as part of well-recognized endocrine tumor syndromes or as familial isolated pituitary adenoma (FIPA). The identification of germline pathogenic variants in the aryl hydrocarbon receptor-interacting protein (*AIP*) gene in 2006 represented a major advance in the understanding of hereditary pituitary tumorigenesis, establishing *AIP* as the principal susceptibility gene for FIPA [1,2].

The genetic basis of pituitary disorders encompasses both genes involved in pituitary development and hormone-producing cell differentiation and genes associated with hereditary pituitary tumor predisposition. An important early milestone was the identification of a mutation in the pituitary-specific transcription factor PIT1, encoded by *POU1F1*, in patients with combined pituitary hormone deficiency, as reported by Pfäffle et al. in 1992 [3]. Although *POU1F1* mutations are primarily associated with congenital hypopituitarism rather than hereditary PitNET predisposition, this discovery contributed to the understanding of the molecular regulation of pituitary function. Subsequent advances identified several genes involved in hereditary pituitary tumor predisposition, including *AIP*, *MEN1*, *CDKN1B*, *PRKAR1A*, and *GPR101*, highlighting the genetic heterogeneity of pituitary disorders.

Subsequent international studies demonstrated that *AIP*-related PitNETs are associated with a distinct clinical phenotype characterized by younger age at diagnosis, predominance of growth hormone (GH)-secreting tumors, larger tumor size, increased invasiveness and a greater need for multimodal treatment compared with *AIP*-negative tumors [2,4]. These findings highlighted the clinical importance of identifying affected patients and their relatives, as early diagnosis may influence therapeutic management and long-term outcomes.

Although germline pathogenic *AIP* variants are uncommon among unselected adult patients with sporadic PitNETs, their prevalence increases substantially in selected clinical settings. The highest diagnostic yield has consistently been reported in patients with FIPA, pituitary gigantism, childhood- or adolescent-onset GH-secreting PitNETs, and young patients presenting with pituitary macroadenomas, particularly those diagnosed before 30 years of age [5,6]. These observations have progressively shifted genetic testing from an empirical approach toward phenotype-driven patient selection based on clinical predictors of carrying a pathogenic *AIP* variant.

Beyond confirming the genetic diagnosis, identification of a pathogenic *AIP* variant has important implications for patient care. It enables genetic counseling, cascade testing of at-risk relatives and clinical surveillance of pathogenic variant carriers, facilitating earlier tumor detection before the development of large invasive lesions. Prospective studies have shown that relatives diagnosed through surveillance programs generally present with smaller and less invasive tumors than clinically presenting carriers, supporting the clinical value of targeted genetic screening [4,6,7].

Despite the increasing availability of germline testing, several challenges remain. Published studies differ considerably with respect to patient selection, age thresholds, tumor subtype distribution, sequencing methodologies, copy-number analysis and, more recently, variant classification according to contemporary ACMG/AMP criteria [7]. These differences contribute to substantial variability in the reported prevalence of pathogenic *AIP* variants and complicate direct comparisons between published cohorts [5,6].

The aim of this review is to critically evaluate the current evidence regarding germline *AIP* testing in patients with PitNETs, focusing on the diagnostic yield across different clinical scenarios, the factors associated with an increased likelihood of identifying pathogenic variants and the practical selection of patients most likely to benefit from genetic evaluation.

## 2. Materials and Methods

This article was designed as a narrative review addressing the clinical indications, diagnostic yield, and interpretation of germline *AIP* testing in patients with pituitary neuroendocrine tumors (PitNETs).

A literature search was performed using PubMed, including MEDLINE-indexed records, to identify relevant publications. Searches were conducted using multiple combinations of the following keywords: “AIP”, “aryl hydrocarbon receptor-interacting protein”, “pituitary adenoma”, “pituitary neuroendocrine tumor”, “familial isolated pituitary adenoma (FIPA)”, “pituitary gigantism”, “acromegaly”, “prolactinoma”, “genetic testing”, and “germline mutation”. Boolean operators (AND and OR) were used to combine search terms. Representative search combinations included (“AIP” OR “aryl hydrocarbon receptor-interacting protein”) AND (“pituitary adenoma” OR “pituitary neuroendocrine tumor” OR “FIPA”), with additional combinations incorporating terms related to genetic testing, diagnostic yield, and variant interpretation. The representative Boolean search strategies are provided in Supplementary Table S1, and the literature identification and narrative selection process is illustrated in Supplementary Figure S1.

Original clinical studies, international multicenter cohorts, systematic reviews, consensus statements, clinical practice guidelines, and other relevant publications written in English and published between January 2006 and April 2025 were considered. Publications were selected based on their relevance to the objectives of the review, with preference given to studies reporting diagnostic yield, genotype–phenotype correlations, clinical indications for germline *AIP* testing, and contemporary approaches to variant interpretation. Additional relevant publications were identified through manual screening of the reference lists of selected articles.

Given the narrative design of the review, no predefined systematic screening protocol or formal risk-of-bias assessment was applied. The available evidence was interpreted qualitatively, considering study design, sample size, cohort characteristics, patient selection, genetic testing methodology, and variant classification criteria. Particular attention was paid to potential referral and ascertainment biases, differences in reported diagnostic yield, and changes in genetic testing technologies and variant classification practices over time. Historical studies were interpreted in the context of current ACMG/AMP recommendations for sequence variant classification when appropriate.

As numerical records of publications identified, screened, excluded, and included were not systematically maintained, these figures could not be reliably reconstructed retrospectively.

ChatGPT (OpenAI, GPT-5.6) was used solely for language editing, stylistic refinement, and reference formatting. It was not used for literature searching or selection, scientific analysis, interpretation of the literature, or formulation of the scientific conclusions.

## 3. Biology and Clinical Phenotype of AIP-Related PitNETs

The aryl hydrocarbon receptor-interacting protein (*AIP*) gene, located on chromosome 11q13.2, encodes a 330-amino acid co-chaperone protein that is highly expressed in the anterior pituitary. Initially identified through its interaction with the aryl hydrocarbon receptor (AHR), *AIP* is now recognized as a multifunctional protein involved in several intracellular signaling pathways through interactions with heat shock protein 90 (HSP90), phosphodiesterase 4A5 (PDE4A5) and other client proteins involved in protein stabilization, intracellular signaling and cell-cycle regulation [1,8].

Although the precise mechanisms underlying *AIP*-associated pituitary tumorigenesis remain incompletely understood, available evidence supports its role as a tumor suppressor gene. Functional studies have demonstrated that pathogenic *AIP* variants impair its anti-proliferative activity, while most *AIP*-associated tumors exhibit loss of heterozygosity, supporting a classical two-hit mechanism of tumorigenesis. Altered cAMP signaling secondary to impaired interaction with PDE4A5, together with dysregulation of additional pathways including ZAC1 and Gαi-mediated signaling, are considered major contributors to pituitary tumor development [9,10].

Beyond its role in cAMP signaling, *AIP* also participates in maintaining protein stability through interactions with HSP90 and several client proteins. Most germline pathogenic variants identified in patients with PitNETs are truncating or missense variants affecting the tetratricopeptide repeat (TPR) domain, which mediates protein–protein interactions. Structural studies have demonstrated that disruption of the C-terminal α-7 helix within the TPR domain markedly impairs binding to client proteins, providing a structural explanation for the pathogenicity of many disease-associated variants [7]. These findings support the concept that defective chaperone function is a central mechanism underlying *AIP*-associated pituitary tumorigenesis.

Consistent with its role as a tumor suppressor gene, tumor development in pathogenic variant carriers usually follows a classical two-hit model, with germline inactivation of one allele followed by somatic loss of the remaining wild-type allele. Loss of heterozygosity has been demonstrated in a substantial proportion of *AIP*-associated PitNETs, although not universally, suggesting that additional genetic and epigenetic events contribute to tumor initiation and progression [9,10]. This biological heterogeneity may partly explain the marked variability in disease penetrance and clinical presentation observed among individuals carrying the same germline pathogenic variant.

Clinically, pathogenic *AIP* variant carriers display a characteristic phenotype that clearly differs from sporadic PitNETs. International cohort studies consistently report younger age at diagnosis, a predominance of GH-secreting tumors, frequent pituitary gigantism, larger tumor size and a higher prevalence of extrasellar invasion [2,4,11]. In addition, *AIP*-positive somatotroph tumors often exhibit reduced responsiveness to first-generation somatostatin receptor ligands and more frequently require multimodal treatment, including repeated surgery and radiotherapy [4]. Nevertheless, these clinical characteristics should not be equated with aggressive PitNETs, as the revised 2025 European Society of Endocrinology guidelines highlight that it remains uncertain whether carriers of germline pathogenic variants have an increased risk of aggressive pituitary tumor behavior [12].

Recognition of this phenotype has become the cornerstone of contemporary patient selection for germline *AIP* testing. Rather than universal germline screening of all PitNETs, current evidence supports a phenotype-driven approach in which the probability of identifying a pathogenic *AIP* variant is highest in patients with GH excess presenting at a young age, pituitary gigantism, large or invasive macroadenomas and a positive family history [5,6,11].

Despite the distinctive phenotype observed in carriers of pathogenic *AIP* variants, these variants account for only a minority of hereditary PitNETs, even among FIPA families. The absence of an identifiable *AIP* variant therefore does not exclude an underlying hereditary predisposition and should prompt consideration of alternative susceptibility genes when clinical suspicion remains high [6,11].

These genotype–phenotype correlations provide the biological rationale for the phenotype-driven strategy currently used to select patients for germline *AIP* testing, which is discussed in the following sections.

**Figure 1:**
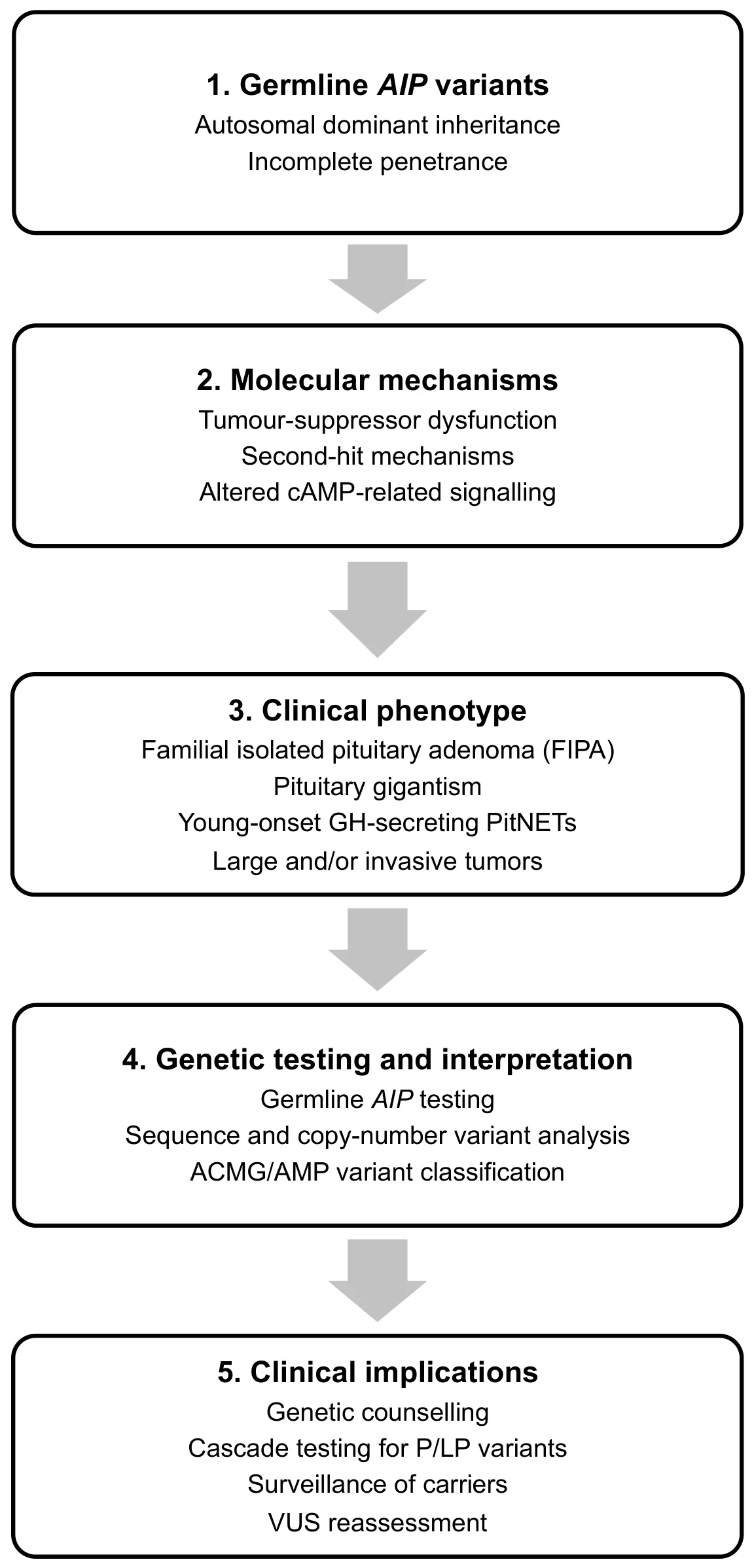
Overview of germline *AIP*-related pituitary neuroendocrine tumors: from molecular mechanisms to clinical implications. The schematic summarizes the hereditary basis of *AIP*-related PitNETs, the main molecular mechanisms implicated in tumor development, characteristic clinical phenotypes, genetic testing and variant interpretation, and implications for patients and their families. The figure represents an author-developed synthesis of the literature. Abbreviations: AIP, aryl hydrocarbon receptor-interacting protein; ACMG/AMP, American College of Medical Genetics and Genomics/Association for Molecular Pathology; cAMP, cyclic adenosine monophosphate;FIPA, familial isolated pituitary adenoma; GH, growth hormone; P/LP, pathogenic/likely pathogenic; PitNET, pituitary neuroendocrine tumor; VUS, variant of uncertain significance.

The genetic basis, molecular mechanisms, characteristic clinical phenotypes, and clinical implications of germline AIP-related PitNETs are summarized in Figure 1.

## 4. Which Patients Should Undergo Germline AIP Testing?

### 4.1. Patients with Familial Isolated Pituitary Adenoma

Familial isolated pituitary adenoma (FIPA) is defined as the occurrence of PitNETs in two or more related individuals in the absence of clinical or genetic evidence of other hereditary endocrine tumor syndromes, particularly multiple endocrine neoplasia type 1 (MEN1) and Carney complex [2]. FIPA may be classified as homogeneous, when affected family members develop the same PitNET subtype, or heterogeneous, when different tumor subtypes occur within the same family [2].

FIPA represents the clinical setting with the highest and most consistently reproducible diagnostic yield for germline *AIP* testing. Across the major international cohorts, pathogenic *AIP* variants have been identified in approximately 15–31% of FIPA families, although the higher estimates originate from earlier studies that pre-dated the ACMG/AMP variant-classification framework and should therefore be interpreted with caution [2,10]. The largest international study, including 216 FIPA families, identified pathogenic *AIP* variants in 37 families (17.1%), confirming *AIP* as the principal susceptibility gene underlying hereditary isolated PitNET predisposition [11].

Beyond establishing disease susceptibility, these studies consistently demonstrated a characteristic clinical phenotype among individuals with pathogenic *AIP* variants. Affected patients are typically diagnosed at a younger age, most frequently develop GH-secreting or somatomammotroph PitNETs, and commonly present with macroadenomas showing invasive growth. In contrast, prolactinomas and clinically non-functioning PitNETs occur less frequently, although both homogeneous and heterogeneous FIPA families have been described [2,10,11].

Importantly, the majority of FIPA families remain *AIP*-negative, highlighting the genetic heterogeneity of hereditary pituitary tumor predisposition. Consequently, a negative *AIP* result should not exclude an inherited syndrome when clinical suspicion remains high. In these situations, broader multigene testing should be considered, particularly when the phenotype overlaps with MEN1, MEN4, Carney complex, X-linked acrogigantism or other hereditary endocrine tumor syndromes [6].

From a practical perspective, germline *AIP* testing should be offered to all patients fulfilling the diagnostic criteria for FIPA, irrespective of the tumor subtype identified in the index case. When a pathogenic variant is detected, cascade genetic testing and clinical surveillance should be recommended for at-risk relatives, as prospective screening has been shown to identify tumors at an earlier stage than clinically presenting disease [11].

### 4.2. Patients with Pituitary Gigantism

Pituitary gigantism is one of the strongest clinical indications for germline *AIP* testing. Unlike adult-onset acromegaly, GH excess occurring before epiphyseal closure is frequently associated with an underlying genetic predisposition, with *AIP* representing the most commonly identified susceptibility gene in non-syndromic cases. 

The largest international collaborative study of pituitary gigantism included 208 patients, of whom 143 underwent genetic testing. Germline pathogenic *AIP* variants or deletions were identified in 42 patients (29.4%), making *AIP* the single most frequent identifiable genetic cause of pituitary gigantism. Importantly, affected patients included both members of FIPA kindreds and apparently sporadic cases, indicating that the absence of a positive family history should not preclude genetic testing [13].

Evidence from earlier studies supports these findings. In a prospective cohort of apparently sporadic acromegaly, Cazabat et al. identified pathogenic *AIP* variants exclusively in young patients with GH-secreting PitNETs; three of the five affected patients presented with pituitary gigantism [14]. Likewise, pediatric cohorts have consistently demonstrated a marked enrichment of germline pathogenic *AIP* variants among children and adolescents with GH- or GH/PRL-secreting PitNETs, whereas pathogenic *AIP* variants are exceptionally rare in isolated corticotroph tumors [15].

From a clinical perspective, pituitary gigantism should prompt early genetic evaluation irrespective of family history. However, *AIP* is not the only genetic cause of childhood GH excess. Very early-onset disease, particularly during infancy, should raise suspicion for X-linked acrogigantism caused by GPR101 duplication, while patients with additional syndromic features should be evaluated using a phenotype-directed multigene panel [6].

Overall, current evidence consistently supports germline genetic testing in all patients with pituitary gigantism, with *AIP* included among the established pituitary predisposition genes. Pituitary gigantism remains one of the highest-yield clinical scenarios for genetic testing and should be regarded as a first-line indication for referral to genetic counseling [6,11,13].

### 4.3. Pediatric Pituitary Neuroendocrine Tumors

Pediatric PitNETs account for a small proportion of all pituitary tumors but are substantially more likely to have an underlying genetic etiology than adult-onset disease. However, the probability of identifying a germline pathogenic *AIP* variant is not uniform across all pediatric PitNET subtypes and depends largely on tumor lineage and clinical presentation.

Current evidence demonstrates that pathogenic *AIP* variants are predominantly associated with childhood-onset GH-secreting or mixed GH/PRL-secreting PitNETs. In the prospective international study by Tichomirowa et al., the overall historical prevalence of *AIP* variants among patients diagnosed before 30 years of age was 11.7%, increasing to 20.5% in pediatric patients (<18 years). Patients carrying pathogenic *AIP* variants predominantly presented with somatotroph and lactotroph macroadenomas, whereas clinically non-functioning tumors were less frequently observed [5]. Similarly, Stratakis et al. identified truncating *AIP* variants in three of six pediatric patients with GH/PRL-secreting PitNETs but found no convincing evidence supporting an association between pathogenic *AIP* variants and isolated pediatric corticotroph tumors [15].

These observations indicate that pediatric age alone should not be considered an isolated indication for germline *AIP* testing. Instead, testing should be prioritized in children and adolescents presenting with GH excess, pituitary gigantism, macroadenomas or a positive family history, all of which substantially increase the likelihood of identifying a pathogenic variant [11,13,16]. Conversely, the currently available evidence does not support routine *AIP* testing in children with isolated corticotroph tumors or other uncommon PitNET subtypes in the absence of additional clinical features suggestive of hereditary disease [15].

Given the relatively high prevalence of hereditary pituitary tumor syndromes in childhood, the diagnostic evaluation should extend beyond *AIP* when clinically appropriate. Patients with very early-onset GH excess, syndromic manifestations or atypical tumor phenotypes should undergo phenotype-directed multigene testing to exclude alternative genetic causes, including GPR101, MEN1, CDKN1B and other susceptibility genes [6].

From a practical perspective, current evidence supports germline genetic testing in pediatric patients with GH-secreting or GH/PRL-secreting PitNETs, with *AIP* included among the genes tested. In contrast, universal *AIP* testing for all pediatric PitNETs is not supported by the available literature and should instead be guided by tumor subtype and the overall clinical phenotype [6].

### 4.4. Young-Onset Sporadic Pituitary Neuroendocrine Tumors

Young age at diagnosis is one of the strongest clinical predictors of a germline pathogenic *AIP* variant and represents a key criterion for selecting patients with apparently sporadic PitNETs for genetic evaluation. However, chronological age alone is insufficient to identify individuals at risk, as the diagnostic yield of *AIP* testing varies considerably according to tumor subtype, tumor size and clinical presentation [5,6,11].

The relationship between age at diagnosis and the likelihood of identifying a germline pathogenic *AIP* variant has been consistently demonstrated across multiple cohorts. In the landmark study by Tichomirowa et al., focused screening of patients with sporadic pituitary macroadenomas diagnosed at or before 30 years of age identified historical *AIP* variants in 11.7% of cases, with the prevalence increasing to 20.5% among pediatric patients. Most pathogenic *AIP* variant carriers harbored GH-secreting or prolactin-secreting macroadenomas, highlighting the importance of combining age with tumor phenotype when selecting candidates for genetic testing [5].

Subsequent studies refined these observations. In the largest international cohort of young-onset PitNETs, Hernández-Ramírez et al. identified pathogenic *AIP* variants in 8.4% of apparently sporadic patients diagnosed at or before 30 years of age, confirming that young age substantially enriches the diagnostic yield compared with unselected adult PitNET populations [11]. More recently, Gaspar et al. evaluated 218 consecutive Portuguese patients with apparently sporadic pituitary macroadenomas diagnosed before the age of 40 years using contemporary ACMG/AMP variant classification. Although rare *AIP* sequence variants were identified in 8.3% of patients, only four individuals (1.8%) harbored pathogenic or likely pathogenic variants. All patients carrying pathogenic or likely pathogenic (P/LP) *AIP* variants had GH-secreting PitNETs diagnosed between 14 and 25 years of age. The prevalence of pathogenic or likely pathogenic variants increased to 3.4% among patients diagnosed before 30 years and to 5.0% among pediatric patients, highlighting the importance of combining age with tumor phenotype when selecting candidates for genetic testing [16]. In another recent cohort, Trofimiuk-Müldner et al. evaluated 131 adult patients with apparently sporadic pituitary macroadenomas and identified germline AIP variants in 5 patients (3.8%), although the pathogenic significance of some identified variants remains uncertain under contemporary classification frameworks [17].

These differences between historical and contemporary studies primarily reflect changes in variant interpretation rather than true differences in disease prevalence. Earlier studies frequently classified rare missense variants as pathogenic, whereas current ACMG/AMP criteria apply more stringent standards for variant classification, resulting in lower but more clinically reliable estimates of diagnostic yield [6].

Taken together, the available evidence indicates that young age should not be considered an isolated indication for germline *AIP* testing. Instead, age should be interpreted together with tumor subtype, macroadenoma status, GH excess, pituitary gigantism and family history in a phenotype-driven approach that maximizes diagnostic yield while minimizing unnecessary genetic testing [6,11].

### 4.5. Patients with Sporadic Somatotroph Pituitary Neuroendocrine Tumors

Somatotroph PitNETs are the tumor subtype most frequently associated with germline pathogenic *AIP* variants. However, the presence of a somatotroph tumor alone does not justify routine genetic testing, as the prevalence of pathogenic *AIP* variants in apparently sporadic acromegaly varies considerably according to patient selection [4,5,6,11].

Several studies have consistently demonstrated that carriers of pathogenic *AIP* variants exhibit a characteristic clinical phenotype. Compared with *AIP*-negative patients, they are typically diagnosed at a younger age and more frequently present with large, invasive somatotroph macroadenomas and pituitary gigantism [4,11,13]. Consequently, the diagnostic yield of germline *AIP* testing is highest among patients with early-onset GH excess, particularly when associated with macroadenomas or a positive family history [6,11].

The importance of careful patient selection is highlighted by the marked differences observed between historical and contemporary screening studies. Early investigations reported frequencies of *AIP* variants considered pathogenic at the time ranging from approximately 3% to 5% in selected cohorts of apparently sporadic acromegaly, with such variants occurring almost exclusively in young patients with macroadenomas [14]. More recently, Gaspar et al. evaluated a consecutive cohort of young patients with sporadic pituitary macroadenomas using contemporary ACMG/AMP variant-classification criteria and identified pathogenic or likely pathogenic *AIP* variants in only four patients (1.8% of the overall cohort). All four had GH-secreting PitNETs diagnosed between 14 and 25 years of age [16]. These findings suggest that the lower prevalence of pathogenic or likely pathogenic *AIP* variants reported in recent studies primarily reflects more stringent variant interpretation rather than a true change in disease epidemiology [6].

Current evidence therefore supports a phenotype-driven approach rather than routine genetic testing for all patients with sporadic somatotroph PitNETs. The combination of young age at diagnosis, GH-secreting macroadenoma, pituitary gigantism and a positive family history identifies the subgroup with the highest probability of harboring a germline pathogenic *AIP* variant, whereas routine testing in patients with typical adult-onset sporadic acromegaly without additional risk factors has a consistently low diagnostic yield [6,11,16].

### 4.6. Patients with Prolactinomas

Prolactinomas represent the second most frequent PitNET subtype identified in patients carrying germline pathogenic *AIP* variants after somatotroph tumors. However, the association between pathogenic *AIP* variants and prolactinomas is considerably weaker than that observed for GH-secreting PitNETs, and the diagnostic yield of genetic testing in apparently sporadic prolactinomas remains low [2,4,5,6,11].

Within FIPA kindreds, prolactinomas occur in both homogeneous and heterogeneous families, although somatotroph tumors remain the predominant phenotype among carriers of pathogenic *AIP* variants [2,10,11]. In contrast, studies evaluating apparently sporadic prolactinomas have shown that pathogenic *AIP* variants are identified almost exclusively in selected high-risk patients, particularly those diagnosed at a young age with macroprolactinomas [5,6,11].

Young age at diagnosis appears to be the strongest predictor of a positive genetic result in patients with prolactinomas. Historical screening studies demonstrated a higher prevalence of *AIP* variants in young patients with macroadenomas than in unselected prolactinoma cohorts, whereas contemporary studies applying ACMG/AMP variant-classification criteria indicate that clinically actionable pathogenic variants are uncommon outside carefully selected populations [5,6,16]. These findings emphasize that patient selection has a greater impact on diagnostic yield than tumor subtype alone.

Current evidence therefore does not support routine germline *AIP* testing in all patients with prolactinomas. Instead, testing should be considered in patients with early-onset macroprolactinomas, particularly when accompanied by a positive family history or other clinical features suggestive of hereditary pituitary tumor predisposition [6,11]. Conversely, routine genetic testing in patients with typical adult-onset microprolactinomas is not justified because of the consistently low prevalence of pathogenic *AIP* variants [6,16].

The principal clinical studies evaluating germline *AIP* testing across different clinical settings are summarized in Table 1. Evidence-based recommendations for patient selection are presented in Table 2.

**Table 1:** Major clinical studies evaluating germline *AIP* testing across different clinical settings.

Study	Population/Inclusion Criteria	Tested Unit (*n*)	Reported *AIP* Findings	Variant-Classification Framework/Key Qualification
Vierimaa et al. 2006 [1]	Northern Finland population-based GH-secreting PitNET series	45 patients	Two *AIP* mutations accounted for 16% overall and 40% among patients diagnosed before 35 years.	Pre-ACMG/AMP; original-author mutation classification
Daly et al. 2007 [2]	FIPA	73 families; 156 affected patients	11/73 families (15.1%) harbored *AIP* mutations; mutation-positive patients were younger and predominantly had somatotroph-lineage macroadenomas.	Pre-ACMG/AMP; original-author mutation classification; family-level yield.
Cazabat et al. 2007 [14]	Apparently sporadic acromegaly	154 patients	AIP mutations detected in 5/154 (3.0% reported by the authors; 3.2% arithmetic proportion); three of five patients counted by the authors presented with pituitary gigantism.	Pre-ACMG/AMP.
Georgitsi et al. 2007 [18]	Multiple European and US screening cohorts, including young acromegaly, unselected acromegaly/PitNETs and *MEN1*-negative cases	460 participants across several cohorts	Nine presumably pathogenic mutations: 2/27 German young acromegaly; 2/36 Finnish young acromegaly; 0/71 Italian acromegaly; 2/113 US PitNETs; 1/122 Polish PitNETs; 1/55 Spanish *MEN1*-negative; 1/36 Dutch *MEN1*-negative.	Pre-ACMG/AMP. The authors regarded the pathogenicity subdivision as preliminary; contemporary reassessment is required.
Leontiou et al. 2008 [9]	FIPA	26 families	9/26 families (34.6%) harbored *AIP* mutations.	Pre-ACMG/AMP; the authors classified these as *AIP* mutations and provided functional characterization.
Buchbinder et al. 2008 [19]	Sporadic functioning and non-functioning PitNETs	110	3/110 patients (2.7%) were reported by the authors as harboring an *AIP* germline mutation.	Pre-ACMG/AMP; original-author mutation classification; all three mutations occurred in non-functioning PitNETs. The reported yield should not be interpreted as contemporary P/LP prevalence without reclassification.
Igreja et al. 2010 [10]	FIPA	64 families	20/64 families (31.3%) with *AIP* mutations.	Pre-ACMG/AMP; the authors used mutation terminology for disease-associated findings and variant terminology for sequence changes; functional evidence informed interpretation.
Occhi et al. 2010 [20]	Italian apparently sporadic acromegaly cohort	131	4/131 (3.1%) reported *AIP* mutations.	Pre-ACMG/AMP; original-author mutation classification; contemporary P/LP yield is uncertain because the clinical significance of some variants requires reassessment.
Stratakis et al. 2010 [15]	Isolated pediatric GH/PRL-secreting PitNETs and isolated Cushing disease; additional familial/syndromic cases assessed separately	6 GH/PRL PitNETs; 74 Cushing disease cases	3/6 patients in the isolated GH/PRL subgroup had reported *AIP* mutations; 1/74 patients with isolated Cushing disease had a reported *AIP* missense mutation (p.Lys103Arg).	Pre-ACMG/AMP. The 50% yield applies only to the very small isolated GH/PRL subgroup. The relevance of the corticotroph-associated missense finding remains unconfirmed.
Tichomirowa et al. 2011 [5]	Apparently sporadic pituitary macroadenomas diagnosed at ≤30 years	163 patients, including 39 pediatric patients	19/163 (11.7%) with germline *AIP*mut; 8/39 (20.5%) in pediatric patients had *AIP*mut.	Pre-ACMG/AMP focused-screening yield; the authors distinguished AIPmut from additional sequence changes of uncertain significance or polymorphism; reported variants should not automatically be interpreted as contemporary P/LP variants.
Cazabat et al. 2012 [21]	Apparently sporadic PitNETs of all histotypes	443 patients	16/443 (3.6%) with *AIP* mutations; 16/222 (7.2%) among patients diagnosed before 40 years.	Pre-ACMG/AMP; original-author mutation classification; none were identified after age 40 in this cohort.
Preda et al. 2014 [22]	Apparently sporadic PitNETs presenting before 40 years	127 patients	4/127 (3.1%) with any *AIP* variant; 2/127 (1.6%) with definite pathogenetic mutations; both definite mutations occurred among 48 patients with acromegaly (4.2%).	Pre-ACMG/AMP; particularly informative because the authors distinguished all detected variants from definite pathogenetic mutations.
Schöfl et al. 2014 [23]	Acromegaly diagnosed at ≤30 years in the German Acromegaly Registry	91 patients	5/91 (5.5%) with *AIP*mut; 3/4 (75%) among patients with a positive family history; approximately 2.3% in apparently sporadic cases.	Pre-ACMG/AMP; *AIP*mut included sequence mutations and gross deletions; family history markedly enriched the yield.
Hernández-Ramírez et al. 2015 [11]	FIPA and apparently sporadic PitNETs with onset at ≤30 years	216 FIPA families; 404 sporadic patients	37/216 FIPA families (17.1%); 34/404 sporadic patients (8.4%) had *AIP*mut.	Study-specific *AIP*mut classification; formal ACMG/AMP five-tier classification was not clearly documented, and historical *AIP*mut status should not be assumed to equal current P/LP classification.
Rostomyan et al. 2015 [13]	Pituitary gigantism	143 genetically tested patients from a total cohort of 208	42/143 (29.4%) with *AIP* mutations or deletions.	Original-author *AIP* mutation/deletion classification; the reported genetic yield predates uniform contemporary P/LP interpretation.
Ramírez-Rentería et al. 2016 [24]	Mexican acromegaly cohort with disease onset before 30 years	71 patients	5/71 (7.0%) harbored germline *AIP* mutations.	Original-author mutation classification; novel findings were considered predicted pathogenic using in silico evidence, while a VUS was reported separately. Formal ACMG/AMP five-tier classification was not documented.
Araujo et al. 2017 [25]	Brazilian apparently sporadic PitNETs: macroadenoma diagnosed at ≤40 years or a PitNET of any size diagnosed at ≤18 years	132 patients	11/132 (8.3%) met the authors’ broad *AIP*mut criterion; 3/132 (2.3%) were classified as pathogenic at the time of the study, while the remaining findings were VUS or likely benign.	ACMG/AMP five-tier classification applied; the reported 8.3% *AIP*mut frequency included VUS and likely benign variants and must not be interpreted as P/LP prevalence.
Caimari et al. 2018 [6]	International referral cohort used to develop an *AIP* risk-category model	1,405 tumor patients in the principal analysis	134/1,405 (9.5%) *AIP* mutations; only variants classified as pathogenic or likely pathogenic were counted as mutations.	P/LP-based classification; only pathogenic or likely pathogenic variants were considered *AIP* mutations by the authors.
Gaspar et al. 2023 [16]	Consecutive apparently sporadic macroadenomas diagnosed before 40 years	218 patients	4/218 P/LP variants (1.8%) overall; 3.4% among patients diagnosed before 30 years and 5.0% among those diagnosed before 18 years.	Explicit ACMG/AMP classification; only pathogenic or likely pathogenic variants were considered causative. All four P/LP carriers had GH-secreting PitNETs diagnosed between 14 and 25 years.
Xiang et al. 2024 [26]	Han Chinese sporadic acromegaly (*n* = 163) and pituitary gigantism (*n* = 18), with onset at ≤45 years	181 patients	10/181 P/LP carriers (5.5%); 15/181 (8.29%) carried any *AIP* variant.	Contemporary P/LP categorization; the reported 12/133 (9.02%) in patients aged ≤30 years refers to all detected *AIP* variants, not exclusively P/LP variants.

Note: Major original studies evaluating germline *AIP* testing across different clinical settings. Diagnostic yield is reported using the denominators and units of analysis specified in the original studies (individual patients or families). *AIP* findings are presented according to the terminology and variant-classification framework used by the original authors. Historical reports of *AIP* mutations should not be considered directly equivalent to contemporary pathogenic/likely pathogenic (P/LP) variant frequencies established using ACMG/AMP criteria. Variants of uncertain significance (VUS) and benign/likely benign variants are distinguished from P/LP variants where reported and should not be included in estimates of clinically actionable diagnostic yield. Some studies include overlapping referral cohorts or previously reported families and should therefore not be pooled quantitatively. Abbreviations: ACMG/AMP, American College of Medical Genetics and Genomics/Association for Molecular Pathology; AIP, aryl hydrocarbon receptor-interacting protein; FIPA, familial isolated pituitary adenoma; GH, growth hormone; MEN1, multiple endocrine neoplasia type 1; P/LP, pathogenic/likely pathogenic; PitNET, pituitary neuroendocrine tumor; PRL, prolactin; VUS, variant of uncertain significance.

**Table 2:** Practical implications of the available evidence for patient selection for germline *AIP* testing.

Clinical Scenario	Evidence from the Literature	Key Supporting Studies	Recommendation Based on Current Evidence
FIPA	*AIP*-associated genetic findings are consistently enriched in FIPA. Historical studies reported *AIP* mutations/AIPmut in approximately 15–31% of families, while current consensus estimates germline pathogenic AIP variants in approximately 15–25% of FIPA kindreds.	Daly 2007 [2]; Igreja 2010 [10]; Hernández-Ramírez 2015 [11]; De Sousa 2026 [27].	Current evidence supports germline genetic testing in patients with FIPA, with *AIP* included among the established pituitary predisposition genes.
Pituitary gigantism	*AIP* mutations or deletions were reported in 42/143 genetically tested patients (29.4%) in the largest international cohort; current consensus similarly identifies pathogenic *AIP* variants as the most frequent known genetic cause, accounting for approximately 30% of pituitary gigantism.	Rostomyan 2015 [13]; De Sousa 2026 [27].	Current evidence supports germline genetic testing in all patients with pituitary gigantism, with *AIP* included among the established pituitary predisposition genes.
Pediatric GH PitNET	*AIP*-associated findings are enriched in pediatric GH- and GH/PRL-secreting PitNETs, particularly in patients with macroadenomas or pituitary gigantism; historical focused cohorts reported high *AIP* mutation/*AIP*mut yields.	Stratakis 2010 [15]; Tichomirowa 2011 [5]; Hernández-Ramírez 2015 [11]; De Sousa 2026 [27].	Current evidence strongly supports germline genetic testing in pediatric GH-secreting or GH/PRL-secreting PitNETs, with *AIP* included among the genes tested.
Young sporadic macroadenoma	Diagnostic yield is enriched in young patients but depends strongly on phenotype and variant-classification framework. Tichomirowa 2011 reported *AIP*mut in 11.7% of patients diagnosed at ≤30 years, whereas Gaspar 2023 identified P/LP AIP variants in 1.8% of the overall cohort and 3.4% of patients diagnosed before 30 years using contemporary ACMG/AMP classification.	Tichomirowa 2011 [5]; Caimari 2018 [6]; Gaspar 2023 [16].	Consider germline genetic testing when young age is accompanied by a high-risk phenotype, particularly GH excess, macroadenoma, pituitary gigantism or a positive family history.
Adult sporadic acromegaly	The diagnostic yield of germline *AIP* testing is low in typical adult-onset sporadic acromegaly and is concentrated predominantly in younger patients with macroadenomas or other high-risk features.	Cazabat 2007 [14]; Cazabat 2012 [21]; Gaspar 2023 [16].	Routine germline *AIP* testing is not supported in typical adult-onset sporadic acromegaly without additional features suggestive of hereditary disease.
Macroprolactinoma at young age	*AIP*-associated findings are uncommon overall but are enriched in selected patients with young-onset macroprolactinomas, particularly when accompanied by a positive family history or other features of hereditary pituitary tumor predisposition.	Tichomirowa 2011 [5]; Hernández-Ramírez 2015 [11]; De Sousa 2026 [27].	Consider germline genetic testing in young-onset macroprolactinoma, particularly when accompanied by a positive family history or other high-risk clinical features.
Corticotroph PitNET	No consistent association between germline pathogenic *AIP* variants and isolated corticotroph PitNETs has been established.	Stratakis 2010 [15].	Routine germline *AIP* testing is not supported for isolated corticotroph PitNETs in the absence of other features suggesting hereditary disease.

Note: Recommendations presented in this table synthesize evidence from the original studies together with contemporary consensus guidance. Historical “mutation” or “AIPmut” frequencies reflect the classification used in the original studies and should not be considered equivalent to contemporary pathogenic/likely pathogenic (P/LP) variant prevalence under ACMG/AMP criteria. Current genetic testing practice increasingly favors multigene panel testing when a hereditary pituitary tumor predisposition is suspected.

### 4.7. Current Recommendations for Germline AIP Testing

The available evidence consistently supports a phenotype-driven rather than a universal approach to germline *AIP* testing. Although *AIP* is the most frequently identified susceptibility gene in hereditary pituitary tumor predisposition, the prevalence of pathogenic variants varies markedly across different clinical scenarios, emphasizing the importance of careful patient selection (Table 1 and Table 2).

The highest diagnostic yield is observed in patients with familial isolated pituitary adenoma (FIPA), pituitary gigantism, and childhood-onset GH-secreting PitNETs. In these clinical settings, germline genetic testing should be routinely considered, with *AIP* included among the established pituitary predisposition genes, irrespective of family history where clinically appropriate [2,11,13,27].

Genetic testing should also be considered in patients with apparently sporadic PitNETs diagnosed at a young age, particularly those presenting before the age of 30 years with GH-secreting macroadenomas or other high-risk clinical features, including pituitary gigantism or a positive family history. Current evidence indicates that combining age with tumor subtype and clinical phenotype provides substantially greater diagnostic accuracy than applying age-based criteria alone [5,6,11,16].

Conversely, routine germline *AIP* testing is not supported in patients with typical adult-onset sporadic acromegaly, microprolactinomas, corticotroph PitNETs or other pituitary tumor subtypes in the absence of additional clinical features suggestive of hereditary disease. In these populations, the prevalence of pathogenic *AIP* variants is consistently low and does not justify universal genetic screening [6,15,16].

An important consideration when interpreting the available literature is the evolution of variant-classification standards. Earlier studies frequently reported higher frequencies of variants considered pathogenic at the time because rare missense variants were often classified as disease-causing. The adoption of ACMG/AMP criteria has resulted in more stringent variant interpretation, lower but more reliable estimates of diagnostic yield, and improved consistency across studies [6,16].

Overall, current evidence, supported by recent systematic reviews and international expert recommendations, favors germline *AIP* testing in carefully selected high-risk patients rather than universal testing of all individuals with PitNETs. A phenotype-driven strategy integrating family history, age at diagnosis, tumor subtype, tumor characteristics, and clinical presentation provides the highest diagnostic yield while avoiding unnecessary testing in low-risk populations (Table 2) [28,29,30,31].

Despite substantial progress in identifying patients most likely to benefit from germline *AIP* testing, several challenges remain. The interpretation of rare genetic variants, the increasing use of multigene panels, and the continuous evolution of variant-classification criteria have significantly influenced both the reported prevalence of pathogenic *AIP* variants and their clinical interpretation. These issues are discussed in the following section.

**Figure 2:**
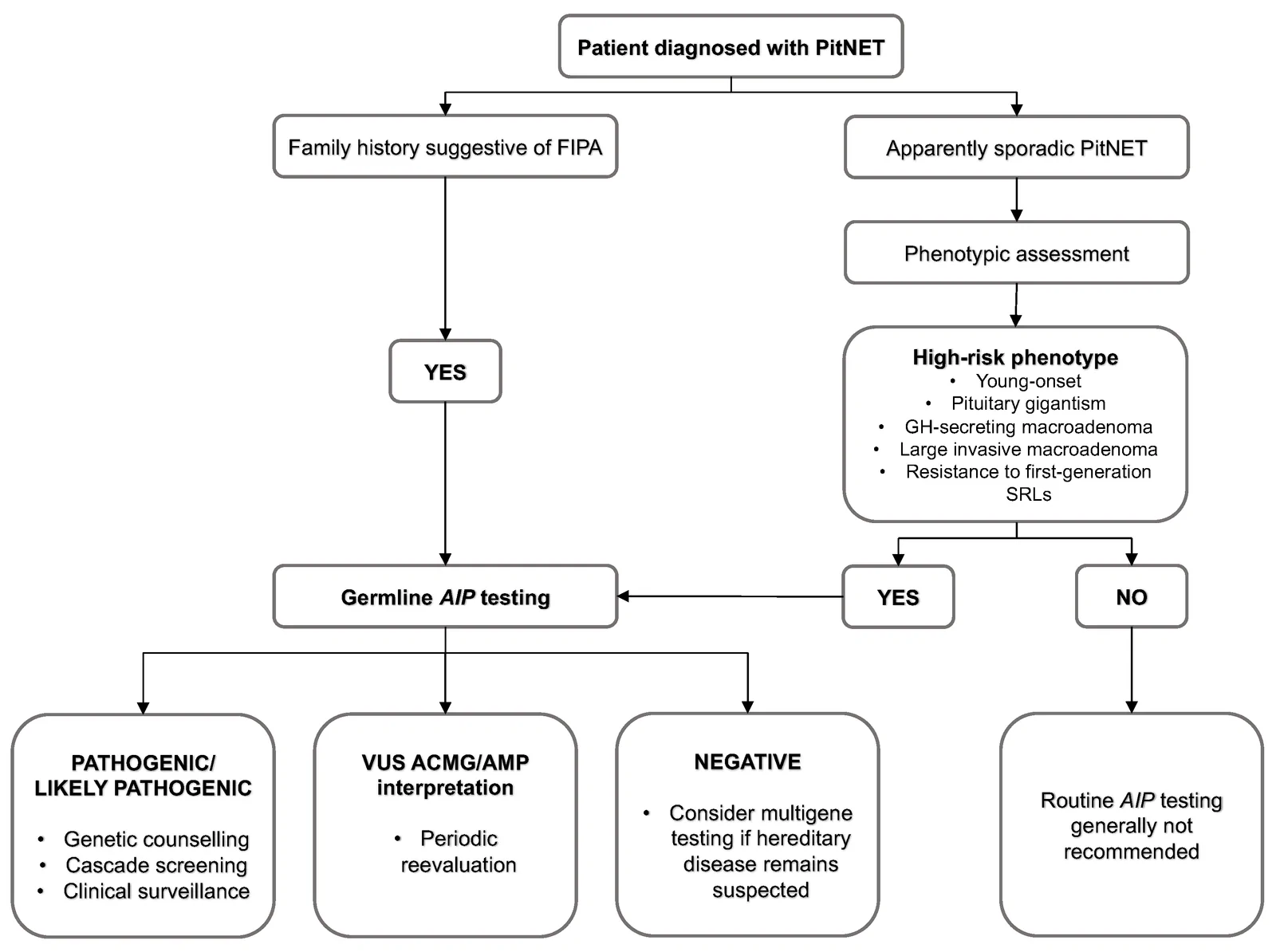
Author-proposed, evidence-informed phenotype-driven clinical algorithm for germline AIP testing in patients with pituitary neuroendocrine tumors (PitNETs). Patients with a family history suggestive of familial isolated pituitary adenoma (FIPA) should be considered for germline AIP testing. In apparently sporadic PitNETs, genetic testing should be considered according to the individual risk profile, particularly in the presence of high-risk clinical features, including young-onset disease, pituitary gigantism, GH-secreting macroadenomas, large invasive macroadenomas, or resistance to first-generation somatostatin receptor ligands (SRLs). The algorithm also summarizes the recommended management according to genetic test results. This algorithm represents a practical synthesis proposed by the authors rather than an officially endorsed society guideline. Abbreviations: *AIP*, aryl hydrocarbon receptor-interacting protein; PitNET, pituitary neuroendocrine tumor; FIPA, familial isolated pituitary adenoma; GH, growth hormone; SRLs, somatostatin receptor ligands; VUS, variant of uncertain significance; ACMG/AMP, American College of Medical Genetics and Genomics/Association for Molecular Pathology.

A practical phenotype-driven approach to patient selection and interpretation of germline *AIP* testing is summarized in Figure 2.

## 5. Challenges and Future Perspectives

### 5.1. Optimizing Patient Selection for Germline AIP Testing

Despite substantial advances in understanding the clinical phenotype associated with germline pathogenic *AIP* variants, defining the optimal indications for genetic testing remains challenging. Although international studies consistently identify young age at onset, familial disease, pituitary gigantism, and GH-secreting macroadenomas as the clinical settings with the highest diagnostic yield, current evidence, including systematic reviews and international consensus statements, supports a targeted, phenotype-driven approach rather than universal screening of all patients with PitNETs [27,28,29,30,31,32]. Nevertheless, the precise age thresholds, clinical selection criteria, and testing algorithms vary slightly between recommendations, reflecting the limited availability of prospective evidence and the heterogeneity of published cohorts.

One of the major challenges is balancing the benefits of early genetic diagnosis against the low prevalence of pathogenic *AIP* variants in unselected PitNET populations. While historical studies frequently recommended genetic testing for all patients diagnosed before 30 or 40 years of age [5,14,20], contemporary cohorts applying rigorous ACMG/AMP variant classification have demonstrated substantially lower diagnostic yields than initially reported [16]. These findings suggest that chronological age alone should no longer be regarded as the principal criterion for genetic testing.

Instead, accumulating evidence supports a phenotype-driven approach integrating multiple clinical variables rather than relying on arbitrary age cut-offs. The probability of identifying a germline pathogenic *AIP* variant increases considerably when young age is accompanied by additional high-risk features, including GH excess, pituitary gigantism, macroadenoma, invasive tumor growth, resistance to first-generation somatostatin receptor ligands, or a family history suggestive of FIPA [4,5,6,11,13,16]. Conversely, the likelihood of detecting a pathogenic variant remains very low in patients with sporadic non-functioning PitNETs or microprolactinomas lacking these clinical characteristics [14,16,20].

This concept is exemplified by the risk stratification model proposed by Caimari et al., which combines age at diagnosis, tumor subtype, family history, and clinical presentation to identify patients with the highest probability of harboring a germline pathogenic *AIP* variant [6]. Although the model demonstrated excellent discriminatory performance within an international referral cohort, external validation in independent and ethnically diverse populations is still lacking. Furthermore, referral cohorts are inherently enriched for hereditary disease and may therefore overestimate diagnostic performance compared with unselected endocrine practice.

However, phenotype-driven testing should not be regarded as an absolute exclusion strategy. Given the incomplete penetrance and variable expressivity of germline *AIP* variants, some carriers may lack characteristic clinical features or an apparent family history. Consequently, although phenotype-based criteria can improve the prioritization of genetic testing, they may fail to identify a proportion of pathogenic variant carriers, and individualized testing should remain an option when clinical suspicion persists.

The cost-effectiveness of extending germline AIP testing to all patients with PitNETs remains uncertain, as formal economic evaluations comparing universal screening with phenotype-driven testing are lacking. Beyond sequencing costs, implementation requires resources for genetic counselling, variant interpretation, and cascade testing of relatives. Although current recommendations generally favor targeted genetic evaluation, differences in testing criteria, reimbursement policies, and access to molecular diagnostics may influence clinical practice across healthcare systems [27]. These considerations support phenotype-informed patient selection as a pragmatic strategy, particularly in resource-limited settings, while highlighting the need for dedicated cost-effectiveness studies.

Future strategies should move beyond simple demographic criteria towards integrated clinical prediction models incorporating phenotype, imaging characteristics, family history, and molecular data. The recent introduction of multigene sequencing panels further highlights the need to reconsider patient selection, as broader testing may identify pathogenic variants in genes other than *AIP* among carefully selected patients with young-onset or atypical PitNETs [33]. At the same time, current expert resources continue to emphasize that phenotype-driven selection remains the most appropriate strategy for routine clinical practice until prospective validation of broader testing approaches becomes available [27,28]. Prospective multicenter studies using standardized phenotyping, contemporary ACMG/AMP variant interpretation, and harmonized reporting will be essential to establish evidence-based testing algorithms that maximize diagnostic yield while avoiding unnecessary investigations and inappropriate interpretation of low-risk variants.

### 5.2. Challenges in Variant Interpretation

Accurate interpretation of germline *AIP* variants has become one of the principal challenges in hereditary PitNET diagnostics. Much of the published literature on *AIP* testing predates the introduction of the ACMG/AMP standards for sequence variant interpretation, and consequently many historical studies reported mutation frequencies based on classification systems that differed substantially from those currently used in clinical practice [7]. As a result, the prevalence of pathogenic *AIP* variants reported in earlier cohorts cannot be directly compared with contemporary studies applying standardized variant classification.

This issue is illustrated by several historical screening studies summarized in Table 1. Buchbinder et al. reported germline *AIP* mutations in three of 110 patients with sporadic PitNETs, whereas Occhi et al. reported *AIP* mutations in four of 131 patients with apparently sporadic acromegaly [18,19]. However, both studies preceded the implementation of the ACMG/AMP framework, and the clinical significance of some reported variants has subsequently been questioned. Consequently, the overall number of reported *AIP* variants should not be interpreted as equivalent to the contemporary prevalence of pathogenic or likely pathogenic variants.

The impact of rigorous variant classification is clearly demonstrated by more recent studies. In a Portuguese cohort of young-onset sporadic pituitary macroadenomas, Gaspar et al. identified rare *AIP* variants in several patients, yet only four fulfilled ACMG/AMP criteria for pathogenicity or likely pathogenicity, resulting in a clinically relevant diagnostic yield of only 1.8% [16]. Similarly, recent reassessment of the frequently reported *AIP* p.Arg304Gln variant concluded that the available genetic, population, and functional evidence supports its reclassification as likely benign rather than disease-causing [32]. This example illustrates how variant reinterpretation may substantially influence estimates of the prevalence of pathogenic *AIP* variants, cascade testing, and long-term clinical management.

These observations have important implications for routine clinical practice. Variants of uncertain significance should not be used to establish a molecular diagnosis, guide treatment decisions, or initiate predictive testing in unaffected relatives. Instead, classification should integrate population frequency, segregation data, computational prediction, functional evidence, and continuously updated public databases according to ACMG/AMP recommendations [7]. Because variant interpretation evolves over time, laboratories should implement periodic reassessment of previously reported variants and establish mechanisms to communicate clinically significant reclassifications to both clinicians and affected families.

Future efforts should focus on developing *AIP*-specific variant interpretation criteria, expanding functional validation of missense variants, and improving international data sharing through curated databases. Functional assays have already demonstrated their value in discriminating pathogenic from non-pathogenic missense variants and are expected to become an increasingly important complement to ACMG/AMP classification, particularly for variants that remain classified as VUS based on current evidence [34]. Such initiatives would reduce inter-laboratory variability, facilitate more consistent classification of rare variants, and ultimately improve the clinical utility of germline *AIP* testing.

As variant interpretation becomes increasingly standardized, the next challenge will be determining how *AIP* testing should be integrated within broader multigene testing strategies for hereditary PitNETs.

### 5.3. Beyond Single-Gene AIP Testing

The increasing availability of next-generation sequencing (NGS) has substantially changed the diagnostic approach to hereditary PitNETs. While *AIP* remains the most frequently implicated gene in FIPA and young-onset somatotroph tumors, several additional susceptibility genes have now been identified, including *MEN1*, *CDKN1B*, *PRKAR1A*, *GPR101*, and genes associated with the succinate dehydrogenase (*SDHx*) complex. Consequently, genetic evaluation is progressively shifting from sequential single-gene analysis towards targeted multigene panels in carefully selected patients [33].

Genetic testing methodologies have also evolved substantially over the period covered by this review. Earlier studies predominantly relied on Sanger sequencing of *AIP* coding regions and splice junctions, whereas contemporary approaches increasingly employ next-generation sequencing (NGS), either through targeted single-gene analysis or multigene panels. Importantly, sequence-based methods alone may fail to identify exon-level or whole-gene deletions and duplications, requiring complementary copy-number analysis, such as multiplex ligation-dependent probe amplification (MLPA) or validated NGS-based approaches [33,35]. Consequently, differences in sequencing coverage, copy-number variant detection, and variant interpretation standards should be considered when comparing the diagnostic yield reported by historical and contemporary cohorts.

Multigene testing offers several potential advantages over isolated *AIP* analysis. Simultaneous evaluation of multiple susceptibility genes increases diagnostic efficiency, particularly in patients with atypical clinical presentations, overlapping hereditary syndromes, or negative *AIP* testing despite a strong clinical suspicion of genetic predisposition. In addition, panel-based testing may reduce the time required to establish a molecular diagnosis while avoiding repeated sequential investigations [33,35].

However, broader genetic testing also introduces important challenges. As the number of genes included in diagnostic panels increases, so does the likelihood of identifying variants of uncertain significance and incidental findings unrelated to the patient’s presenting phenotype. The clinical interpretation of these variants frequently remains uncertain, particularly for genes with limited genotype–phenotype data or incomplete penetrance. Therefore, multigene testing should always be accompanied by appropriate pre-test genetic counseling and careful interpretation within the clinical context, rather than being considered a universal first-line strategy for all patients with PitNETs [7].

At present, isolated *AIP* testing remains appropriate in patients with a highly characteristic phenotype, such as FIPA, pituitary gigantism, or early-onset GH-secreting macroadenomas. In contrast, multigene panels may provide greater clinical value in patients with syndromic features, atypical tumor phenotypes, multiple endocrine neoplasms, or negative *AIP* testing despite persistent suspicion of hereditary disease [33,35]. As sequencing technologies continue to evolve and genotype–phenotype correlations become better defined, broader genomic approaches are expected to complement rather than replace phenotype-driven patient selection.

Ultimately, the challenge is no longer deciding whether to perform genetic testing, but selecting the right patients, the appropriate testing strategy, and accurately interpreting the results within their clinical context.

### 5.4. Future Research Priorities

Despite considerable progress in understanding the genetic basis of hereditary PitNETs, several important questions remain unanswered. One of the principal limitations of the current literature is the predominance of retrospective referral cohorts, which are inherently enriched for patients with aggressive phenotypes or a strong suspicion of hereditary disease. Consequently, the true prevalence of germline pathogenic *AIP* variants in unselected populations remains uncertain, and direct comparisons between published studies are often limited by differences in patient selection, genetic testing strategies, and variant interpretation [6,11,16].

Another important priority is the harmonization of variant interpretation. The implementation of ACMG/AMP recommendations has substantially improved the consistency of variant classification; however, interpretation of rare missense variants and variants of uncertain significance remains challenging. Expanding functional assays, improving segregation analyses, and increasing international data sharing through curated variant databases will be essential to reduce inter-laboratory variability and facilitate more consistent clinical decision-making [7,32,34].

Future studies should also evaluate the role of multigene testing in carefully selected patients with suspected hereditary PitNETs. Although targeted *AIP* analysis remains appropriate in patients with a characteristic clinical phenotype, broader NGS panels may improve diagnostic yield in individuals with atypical presentations or overlapping hereditary syndromes. Prospective studies comparing phenotype-driven single-gene testing with panel-based approaches are needed to determine the most effective and cost-efficient diagnostic strategy [27,33].

Finally, greater international collaboration will be essential to establish robust genotype–phenotype correlations and improve evidence-based recommendations for genetic testing. Such collaborative efforts will be critical for transforming phenotype-driven genetic testing into truly evidence-based precision medicine for patients with PitNETs and their families.

Future guideline updates will likely require incorporation of contemporary ACMG/AMP variant interpretation together with evidence generated from prospective multicenter cohorts evaluating phenotype-driven genetic testing strategies.

Since completion of the literature search, the recently published Pituitary Society International Consensus Statement on genetic testing in pituitary adenomas has further reinforced a phenotype-driven approach to germline testing. The consensus recommendations are broadly consistent with the evidence synthesized in this review, supporting genetic evaluation in carefully selected patients based on age at diagnosis, family history, tumor subtype, and clinical presentation, while emphasizing multidisciplinary genetic counseling and standardized variant interpretation. Importantly, while the present review focuses specifically on germline *AIP* testing, the 2026 consensus places *AIP* within a broader genetic evaluation framework, supporting multigene testing when clinically indicated rather than an exclusively single-gene approach [27].

### 5.5. Limitations of the Review

This review has several methodological limitations related to its narrative design. Literature selection was not conducted according to a predefined systematic screening protocol, and no formal risk-of-bias assessment was performed. Consequently, potential selection bias cannot be excluded, and the findings should be interpreted as a clinically oriented narrative synthesis rather than a systematic evaluation of the available evidence.

## 6. Conclusions

Germline *AIP* testing has become an essential component of the diagnostic evaluation of selected patients with PitNETs, providing important implications for patient management, family screening, and long-term surveillance. Over the past two decades, accumulating evidence has demonstrated that the likelihood of identifying a pathogenic *AIP* variant is not uniform across all pituitary tumors but is strongly influenced by the clinical phenotype. Patients with FIPA, pituitary gigantism, and young-onset GH-secreting macroadenomas consistently show the highest diagnostic yield, whereas routine testing of unselected sporadic PitNETs has limited clinical value.

The introduction of standardized ACMG/AMP variant interpretation has substantially improved the clinical reliability of genetic testing while also highlighting the need to reinterpret historical studies performed before the implementation of contemporary classification criteria. Consequently, previously reported frequencies of *AIP* variants considered pathogenic should be interpreted with caution, as some variants initially considered disease-causing may no longer meet current diagnostic standards. This evolution underscores that the clinical utility of genetic testing depends not only on identifying sequence variants but also on their accurate interpretation within an appropriate clinical context.

Current evidence, supported by recent systematic reviews and international consensus recommendations, favors a phenotype-driven approach to germline *AIP* testing that integrates age at onset, tumor subtype, family history, and clinical presentation rather than isolated demographic criteria. As next-generation sequencing becomes increasingly accessible, multigene testing is expected to complement targeted *AIP* analysis in carefully selected patients with suspected hereditary PitNETs. However, broader genetic testing should be accompanied by rigorous variant interpretation, appropriate genetic counseling, and multidisciplinary clinical assessment.

Future collaborative research incorporating standardized phenotyping, harmonized variant classification, and prospective multicenter cohorts will be essential to refine testing algorithms, improve genotype–phenotype correlations, and optimize the clinical implementation of precision medicine in patients with hereditary PitNETs and their families.

Ultimately, the challenge is no longer determining whether germline *AIP* testing should be performed, but identifying the right patients, selecting the most appropriate testing strategy, and translating genetic findings into clinically meaningful decisions for patients and their families.

## Data Availability

No new data were created or analyzed in this study. Data sharing is not applicable to this article.

## Supplementary Materials

The following supporting information can be downloaded at https://www.mdpi.com/article/10.3390/diagnostics16213471/s1: Supplementary Table S1: Representative Boolean search strategies for literature identification; Supplementary Figure S1: Illustrative overview of the literature identification and narrative selection process.
