# Supplementary material for: Germline AIP Testing in Pituitary Neuroendocrine Tumors: Diagnostic Yield, Variant Interpretation, and a Phenotype-Driven Clinical Approach—A Narrative Review
Source: Diagnostics (Basel). 2026 Oct 24;16(21):3471. doi: 10.3390/diagnostics16213471 (PMC13761486; doi:10.3390/diagnostics16213471)

Table S1. Representative Boolean search strategies for literature identification

Search strategy	Full Boolean search string
**Search 1. AIP-related PitNETs and clinical phenotypes**	(AIP[Title/Abstract] OR "aryl hydrocarbon receptor-interacting protein"[Title/Abstract]) AND ("Pituitary Neoplasms"[MeSH Terms] OR "pituitary adenoma"[Title/Abstract] OR "pituitary adenomas"[Title/Abstract] OR PitNET*[Title/Abstract] OR "pituitary neuroendocrine tumor"[Title/Abstract] OR "pituitary neuroendocrine tumors"[Title/Abstract] OR FIPA[Title/Abstract] OR "familial isolated pituitary adenoma"[Title/Abstract] OR "pituitary gigantism"[Title/Abstract] OR acromegaly[Title/Abstract] OR prolactinoma*[Title/Abstract]) AND (germline[Title/Abstract] OR "Genetic Testing"[MeSH Terms] OR genetic*[Title/Abstract] OR variant*[Title/Abstract] OR mutation*[Title/Abstract]) AND ("2006/01/01"[Date - Publication] : "2025/04/30"[Date - Publication]) AND english[lang]
**Search 2. Diagnostic yield and variant interpretation**	(AIP[Title/Abstract] OR "aryl hydrocarbon receptor-interacting protein"[Title/Abstract]) AND ("Pituitary Neoplasms"[MeSH Terms] OR "pituitary adenoma"[Title/Abstract] OR "pituitary adenomas"[Title/Abstract] OR PitNET*[Title/Abstract] OR "pituitary neuroendocrine tumor"[Title/Abstract] OR "pituitary neuroendocrine tumors"[Title/Abstract] OR FIPA[Title/Abstract] OR "familial isolated pituitary adenoma"[Title/Abstract] OR "pituitary gigantism"[Title/Abstract] OR acromegaly[Title/Abstract] OR prolactinoma*[Title/Abstract]) AND ("diagnostic yield"[Title/Abstract] OR penetrance[Title/Abstract] OR prevalence[Title/Abstract] OR "variant interpretation"[Title/Abstract] OR ACMG[Title/Abstract] OR "pathogenic variant"[Title/Abstract] OR "pathogenic variants"[Title/Abstract] OR "variant of uncertain significance"[Title/Abstract] OR VUS[Title/Abstract]) AND ("2006/01/01"[Date - Publication] : "2025/04/30"[Date - Publication]) AND english[lang]

Abbreviations: AIP, aryl hydrocarbon receptor-interacting protein; FIPA, familial isolated pituitary adenoma; PitNET, pituitary neuroendocrine tumor; ACMG, American College of Medical Genetics and Genomics; VUS, variant of uncertain significance.
Note: The search strategies summarize the principal Boolean combinations used to identify publications relevant to the objectives of this narrative review. Additional relevant studies were identified through manual screening of reference lists.

Figure S1. Illustrative overview of the literature identification and narrative selection process. Publications were identified through PubMed searches and manual screening of relevant reference lists. Literature was selected according to its relevance to germline *AIP* testing in PitNETs. The available evidence was interpreted qualitatively, considering study design, cohort characteristics, genetic testing methodology, diagnostic yield, and variant classification. The diagram illustrates the methodological approach used for this narrative review.

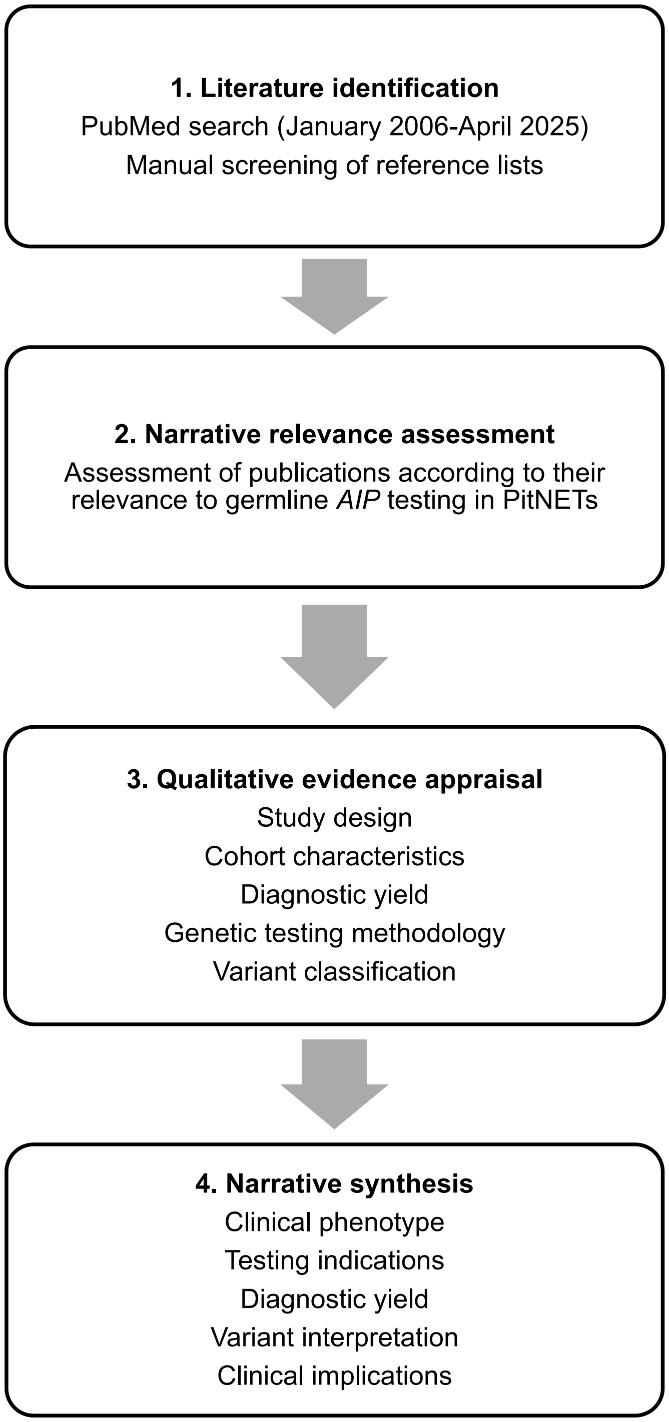

Supplement: Supplementary file 1 [file diagnostics-16-03471-s001.zip › diagnostics-4571616-supplementary.pdf]
